# Exhaled breath profiling for diagnosing acute respiratory distress syndrome

**DOI:** 10.1186/1471-2466-14-72

**Published:** 2014-04-26

**Authors:** Lieuwe DJ Bos, Marcus J Schultz, Peter J Sterk

**Affiliations:** 1 Department of Intensive Care Medicine, Academic Medical Center, University of Amsterdam, Meibergdreef 9, G3–228, 1105 AZ Amsterdam, The Netherlands; 2 Department of Respiratory Care, Academic Medical Center, University of Amsterdam, Amsterdam, The Netherlands

**Keywords:** ARDS, Exhaled breath, Electronic nose, Volatile organic compound, Sensitivity and specificity

## Abstract

**Background:**

The acute respiratory distress syndrome (ARDS) is a common, devastating complication of critical illness that is characterized by pulmonary injury and inflammation. The clinical diagnosis may be improved by means of objective biological markers. Electronic nose (eNose) technology can rapidly and non–invasively provide breath prints, which are profiles of volatile metabolites in the exhaled breath. We hypothesized that breath prints could facilitate accurate diagnosis of ARDS in intubated and ventilated intensive care unit (ICU) patients.

**Methods:**

Prospective single-center cohort study with training and temporal external validation cohort. Breath of newly intubated and mechanically ventilated ICU-patients was analyzed using an electronic nose within 24 hours after admission. ARDS was diagnosed and classified by the Berlin clinical consensus definition. The eNose was trained to recognize ARDS in a training cohort and the diagnostic performance was evaluated in a temporal external validation cohort.

**Results:**

In the training cohort (40 patients with ARDS versus 66 controls) the diagnostic model for ARDS showed a moderate discrimination, with an area under the receiver–operator characteristic curve (AUC–ROC) of 0.72 (95%–confidence interval (CI): 0.63-0.82). In the external validation cohort (18 patients with ARDS versus 26 controls) the AUC–ROC was 0.71 [95%–CI: 0.54 – 0.87]. Restricting discrimination to patients with moderate or severe ARDS versus controls resulted in an AUC–ROC of 0.80 [95%–CI: 0.70 – 0.90]. The exhaled breath profile from patients with cardiopulmonary edema and pneumonia was different from that of patients with moderate/severe ARDS.

**Conclusions:**

An electronic nose can rapidly and non–invasively discriminate between patients with and without ARDS with modest accuracy. Diagnostic accuracy increased when only moderate and severe ARDS patients were considered. This implicates that breath analysis may allow for rapid, bedside detection of ARDS, especially if our findings are reproduced using continuous exhaled breath profiling.

**Trial registration:**

NTR2750, registered 11 February 2011.

## Background

The acute respiratory distress syndrome is a common, devastating complication of critical illness that is characterized by bilateral protein rich pulmonary edema due to injury and inflammation of the lung. A valid and reliable diagnosis of ARDS is considered essential for clinical management and to facilitate enrolment of consistent patient phenotypes into clinical trials [1]. Presently, a new and improved consensus definition of ARDS is used that is based on clinical, radiological and physiological criteria [1]. These criteria are highly suitable for epidemiological studies but only show a moderate correlation with post–mortem pathological findings [2]. ARDS can be mistaken for pneumonia (uni–lateral edema, infection and inflammation) or cardiogenic pulmonary edema (CPE) (low–protein edema due to hydrostatic pressure), and vice versa [2,3]. Thus, there is need for objective markers to group phenotypes more consistently [4].

Use of biological markers could improve the diagnostic process of ARDS since such markers may change before the clinical criteria of ARDS are met [5]. It can be argued that biological markers from lung tissue contain more relevant biochemical information for ARDS diagnosis than plasma markers [6-9]. Exhaled breath contains hundreds of volatile organic compounds (VOCs) that are produced with diverse infectious and inflammatory processes, both in the lung and elsewhere in the body [10-15]. Previous studies of biological markers in the breath of critically ill patients focussed on exhaled breath condensate [16-20]. However, direct analysis of volatile metabolites in the gas phase is also available now [21,22]. This has many advantages, as samples do not require extensive pre–processing, analysis is rapid and may be performed continuously using novel technologies [23].

We hypothesized that VOCs could be used to accurately diagnose and classify ARDS in intubated and ventilated intensive care unit (ICU) patients. The secondary objectives were to investigate the influence of ARDS severity and the underlying causal factor (i.e., pulmonary or non–pulmonary) on diagnostic accuracy. Thirdly, we aimed to investigate the classification of uncomplicated pneumonia and CPE by exhaled breath analysis. Here we focus on exhaled breath profiling (so–called ‘breath prints’) using a electronic Nose (eNose) technology that relies on cross-reactive sensors, meaning that each sensor is responsive to a variety of VOCs [24,25].

## Methods

### Design, subjects and settings

This was a prospective single centre cohort study. All patients admitted to the ICU, with the exception of cardiopulmonary surgery patients, were screened. The only inclusion criterion was mechanical ventilation within the first 24 hours of ICU-admission. Exclusion criteria were (1) previous ICU admission or mechanical ventilation, (2) logistic problems or (3) explicit objection to research by the family.

### Ethical approval and informed consent

The institutional review board of the Academic Medical Center, Amsterdam, The Netherlands, decided that the study did not fulfil all criteria for medical research as stated in the Dutch ‘law on medical research’ because of the non-invasiveness and absence of burden of examining exhaled air (IRB: 10.17.0729). It was judged that exhaled breath could be analyzed without informed consent of the patient. This trial was registered at the Dutch Trial Register (NTR2750).

### Training and validation cohort

The present study strictly adhered to the 25 required items of STARD–guidelines on the investigation of diagnostic accuracy (Additional file 1: Table S1) [26]. During three inclusion periods of ~ 3 months, between January 2011 and February 2012, newly admitted ICU–patients were screened during weekdays. Patients included in the first 2 periods were used in the training cohort; patients included in the last period served as a temporal external validation cohort [27].

### Sample size calculation

Based on a pilot study the estimated sensitivity of exhaled breath profiling for discriminating the two extremes, definite ARDS and definite control patients, was 96.5% [28]. Assuming a prevalence of 50%, an alpha of 0.05 and a 95% confidence interval, the predicted sample size was 104 for the training cohort [29]. A validation cohort half the size of the training cohort was included, according to recommendations on design and analysis of metabolomics studies [30].

### Clinical diagnosis of ARDS

A team of trained clinical research fellows prospectively scored the presence of ARDS [31], which was later re–evaluated according to the new Berlin definition that included the separation in mild, moderate and severe ARDS [1]. Importantly, the assessors were always blind for the eNose signal. All observers were trained on several occasions before the start of the study. All assessors had attended meetings in which clinical case vignettes were discussed and had at least 6 months of work experience [32].

### Competing diagnoses

The diagnosis of community– or hospital–acquired pneumonia consisted of adapted Center for Disease Control–criteria and a post–hoc likelihood of infection was scored (none, possible, probable or proven; see Additional file 1: Table S2) [32,33]. In contrast to ARDS, the diagnosis of CPE required that the findings (acute onset, bilateral infiltrates and PaO2/FiO2 ratio < 300) were fully explained by cardiac dysfunction based on echocardiography [1].

### Exhaled breath profiling

**Figure 1:**
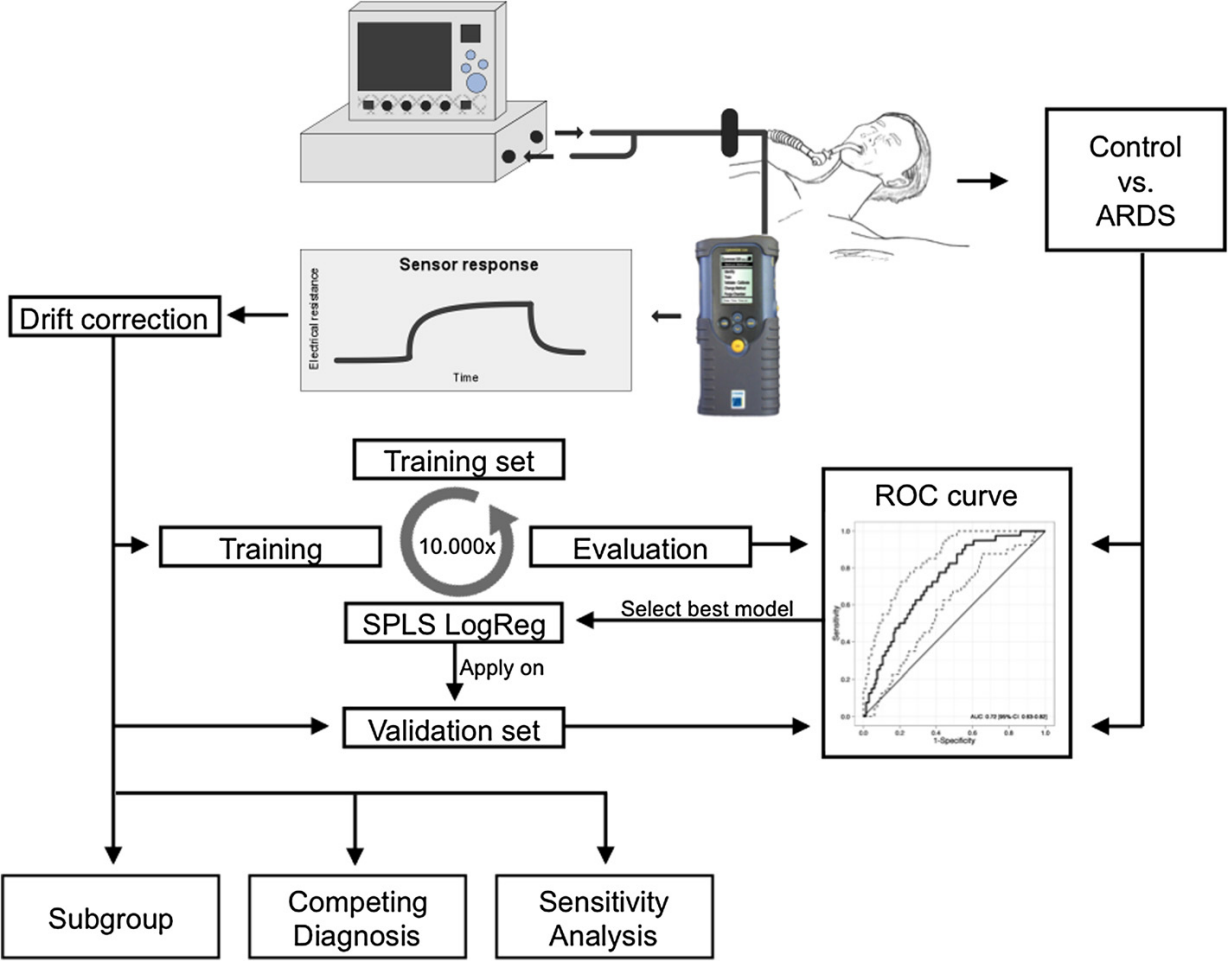
**Sample collection and data analysis.** Exhaled breath was sampled and analyzed using an electronic nose with a side–stream connection distal from the endotracheal tube. This resulted in a response for the 32 polymer sensors in the nano–composite sensor array. The eNose was trained using sparse–partial least square (SPLS) logistic regression with 10.000–fold cross–validation. Data from the training cohort was split into a fraction for model building and model evaluation (10 cases and 10 controls). The algorithm that provided the best internally validated diagnostic accuracy, evaluated by the area under the receiver operating characteristics curve (ROC–AUC), was selected for blind testing in the validation cohort and the ROC–AUC with optimal sensitivity and specificity was reported. Differences in the predictive algorithm between different subgroups (severity of disease, pulmonary and non–pulmonary ARDS) were analyzed using non–parametric tests and the ROC–AUC was reported. Furthermore, the ROC–AUC for distinguishing CPE and pneumonia from ARDS and moderate/severe ARDS only was calculated. A sensitivity analysis was performed using logistic regression on comorbidities that are known to influence breath prints, the PaO2/FiO2 ratio, minute volume ventilation and APACHE II and SAPS II scores.

Existing methodology [21] was adapted for the specific situation of breath collection in intubated and ventilated ICU–patients, as reported previously (Figure 1, upper part) [34]. A co–axial tubing system was connected (Universal F2 breathing circuit, Medical product service GmbH, Braunfis, Germany) to a mechanical ventilator (Galileo ventilator, Hamilton, Bonaduz, Switzerland or Servo ventilator, Maquet, Rastatt, Germany) and a heat–moist exchanger (HME, Medisize, Hillegom, the Netherlands) was placed at the end as part of routine practice. A T–piece connector (T–piece; 22 M/22 F with swivel, Medisize, Hillegom, the Netherlands) was placed between the HME and the swivel (Catheter mount, Medisize, Hillegom, the Netherlands). The swivel was connected to the endotracheal tube (Ruschelit safety clear plus, Teleflex medical, Athlone, Ireland). To produce a side–stream flow, the T–piece was mounted with 50 cm bubbling tube (Bubble tubing PHS3/30G 3×5mm 30 m, Medisize, Vantaa, Finland), which was locked with a three–way stop–cock before insertion into the ventilatory circuit. Exhaled breath was collected (approximately 50 ml/min for 1 minute) and led to a portable eNose, the Cyranose 320 (Smith Detections, Pasadena, CA), containing a nano–composite sensor array with 32 polymer sensors. These sensors swell as volatile organic compounds diffuse into the polymer thereby causing a change in the electrical resistance. The relative change in electrical resistance is saved onto an onboard memory and can later be copied to an offline database. A baseline measurement was performed for 30 seconds through a VOC–filter type A1 (North Safety, Middelburg, the Netherlands). Thereafter, exhaled air was collected and analyzed on line for 60 seconds, using two separate Cyranose eNoses. This procedure was repeated. Data from every initial measurement was disregarded in the analysis because of deviant raw data, as recommended by the manufacturer [21]. The index test and reference test were always performed on the same day, within 24 hours after admission and were blinded for each other.

### Sensor drift over time

We determined sensor drift over time [35]. Per inclusion period, this shift was assumed to be linear. Sensor data was corrected for drift over time, per period, by transformation into standardized residuals by linear regression. This is similar to multiplicative correction, but without the usage of a chemical standard [36].

### Group allocation

ARDS patients were classified as cases and used to train and validate a diagnostic algorithm. Control patients did not fulfil the criteria for ARDS, but could have infiltrates on chest radiography or oxygenation problems, and had no or a low likelihood of having pneumonia or CPE (*e.g.* a patients with interstitial lung disease could be in the control group). The trained algorithm was used to predict the probability of group membership in the patients with competing diagnoses (pneumonia and CPE).

### Statistical analysis

Differences between the groups were compared using the Mann–Whitney U or Kruskal–Wallis test for continuous variables and chi–square for categorical variables. Data was summarized using the median and 25–75^th^ percentile for continuous variables and with count and percentage for categorical variables. All analyses were performed in R statistics using the R–studio interface [37]. P–values below 0.05 were considered significant.

The eNose was trained using sparse–partial least square (SPLS) logistic regression with 10.000–fold cross–validation. SPLS analysis is a form of regression that can select predictive variables and limit false discovery in situations were large number of independent variables are investigated in low numbers of individuals [38]. Data from the training cohort was split into a fraction for model building and model evaluation (10 cases and 10 controls). The algorithm that provided the best, robust internally validated diagnostic accuracy, evaluated by the area under the receiver operating characteristics curve (ROC-AUC), was selected for blind testing in the validation cohort and the ROC-AUC with optimal sensitivity and specificity was reported. The process of temporal external validation is required to assess the actual diagnostic accuracy of the eNose for ARDS [30]. To check for over–fitting of the algorithm, the previous steps were 1000 times repeated with permutated group allocation.

Differences in the predictive algorithm between different subgroups (severity of disease, pulmonary and non–pulmonary ARDS) were analyzed using non–parametric tests and the ROC-AUC was reported. Furthermore, the ROC-AUC for distinguishing CPE and pneumonia from ARDS and moderate/severe ARDS only was calculated. A sensitivity analysis was performed using logistic regression on comorbidities that are known to influence breath prints (chronic pulmonary disease and cancer, see Table 1), the PaO2/FiO2 ratio, minute volume ventilation and measures of severity of disease (Acute Physiology and Chronic Health Evaluation (APACHE) II and Simplified Acute Physiology Score (SAPS) II).

**Table 1:** Patient and physiological characteristics of included patients

		**Control (n = 92)**	**ARDS (n = 58)**	**Pneumonia (11)**	**CPE (19)**	**P–value**
Age	*(years)*	64 (50–75)	57 (54–78)	56 (49–62)	71 (63–79)	0.106
Male	*(yes)*	51 (55)	30 (52)	8 (89)	11 (73)	0.327
APACHE II		20 (15–26)	23 (19–29)	20 (16–24)	23 (20–28)	0.013
SAPS II		48 (37–60)	55 (43–67)	49 (37–55)	57 (46–63)	0.013
Admission type	Medical	56 (62)	41 (72)	7 (64)	16 (89)	0.373
	Elective surgery	5 (6)	2 (4)	0 (0)	0 (0)	
	Emergency surgery	29 (32)	14 (25)	2 (11)	4 (36)	
Comorbidities	Asthma	1 (1)	0 (0)	0 (0)	1 (5)	0.606
	COPD	8 (9)	6 (10)	0 (0)	0 (0)	0.773
	Other respiratory	5 (5)	2 (3)	1 (9)	1 (5)	0.290
	Malignancy	7 (7)	13 (22)	1 (9)	1 (5)	0.090
	DM	10 (11)	10 (17)	0 (0)	3 (16)	0.682
Pmax	*(cmH*_*2*_*O)*	16 (11–20)	21 (15–30)	16 (15–24)	24 (19–29)	< 0.001
PEEP	*(cmH*_*2*_*O)*	5 (5–6)	8 (5–10)	5 (5–8.5)	8 (5–10)	< 0.001
Tidal volume	*(ml)*	456 (393–545)	426 (380–494)	482 (451–579)	410 (373–506)	0.345
Minute volume	*(l/min)*	8.5 (7.4–9.1)	11.0 (9.1–13.3)	11.3 (9.8–12.6)	9.6 (7.9–12.1)	< 0.001
PaCO2	*(kPa)*	5.1 (4.6–5.7)	5.4 (4.6–5.9)	4.7 (4.1–5.6)	4.8 (4.6–5.6)	0.452
PaO_2_/FiO_2_	*(mmHg/%)*	311 (234–398)	212 (165–257)	304 (241–447)	242 (176–264)	< 0.001
Leucocytes	*109/ml*	12.9 (10.2–18.2)	13.4 (8.7–19.4)	14.1 (13.6–15.1)	18.0 (16.0–18.9)	0.053
CRP	*mg/ml*	65 (21–129)	144 (74–237)	135 (62–177)	66 (18–110)	0.002
ICU Mortality		16 (18)	20 (35)	0 (0)	6 (33)	0.023

Continuous variables are expressed as median (25^th^ to 75^th^ percentile). Categorical variables are expressed as number (percentage). Differences between groups are tested using Kruskal-Wallis one way analysis of variance or Chi–square test (with Yates’ correction if necessary) and P–values are reported.

APACHE II: Acute Physiology and Chronic Health Evaluation II; ARDS: acute respiratory distress syndrome; CPE: cardiogenic pulmonary edema; PEEP: Positive end–expiratory pressure; Pmax: maximal inspiratory pressure; SAPS: Simplified Acute Physiology Score.

## Results

### Subjects

**Figure 2:**
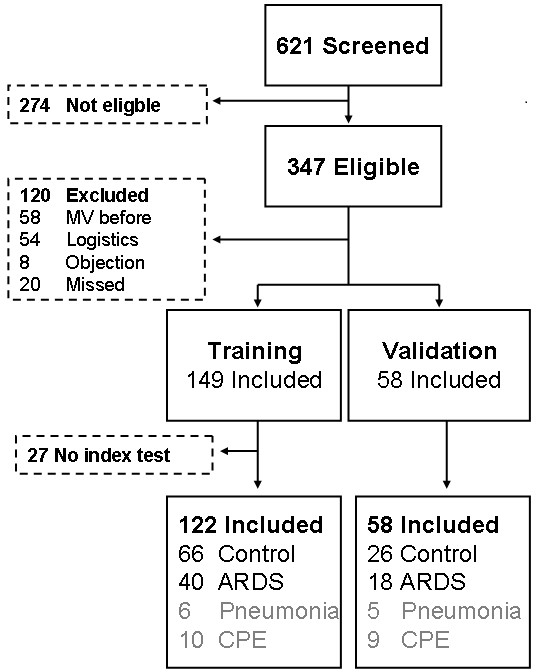
**Flow of patient inclusion.** ARDS: acute respiratory distress syndrome; CPE: cardiogenic pulmonary edema; MV: mechanical ventilation.

Six hundred twenty–one patients were screened, of whom 274 were not eligible and 120 met exclusion criteria (see Figure 2). Thus, 207 patients were included. Exhaled breath profiles were not obtained because of technical problems in 27 patients, leaving 180 patients for analysis. No adverse events were reported during or shortly after breath collection. Fifty–eight (32%) patients fulfilled the definition for ARDS [1], 35 patients were classified as having mild ARDS, and 22 and 1 patient as moderate and severe ARDS, respectively. 92 (51%) patients did not fulfil the definition for ARDS; these patients served as control patients. Competing diagnoses were pneumonia (11 patients) and CPE (19 patients). None of the control patients progressed towards ARDS during the first three days of ICU–admission. Table 1 shows baseline characteristics and respiratory parameters.

### Sensor drift

The sensor signal of the eNoses demonstrated drift over the three periods and within the second period (Additional file 1: Figure S1A). After transformation into standardized residuals by linear regression, these trends disappeared (Additional file 1: Figure S1B).

### Training and internal validation

SPLS logistic regression resulted in the selection of 7 sensors (sensors 4, 8, 9, 11, 16, 28 and 30), the regression coefficients of which can be found in Additional file 1: Table S3. The AUC–ROC for ARDS in the model development cohort was 0.73 (95%–confidence interval (CI): 0.62 – 0.84). Internal validation gave an AUC–ROC for ARDS of 0.71 (CI: 0.47 – 0.95). The diagnostic accuracy for the complete training cohort can be found in Table 2, together with the optimal sensitivity and specificity.

**Table 2:** Diagnostic accuracy of electronic nose analysis

**Cohort**	**Comparison**	**ROC-AUC**	**Specificity**	**Sensitivity**
Training	ARDS vs. Control	0.72 (0.63-0.82)	42%	95%
External validation	ARDS vs. Control	0.71 (0.54-0.87)	50%	89%
In-set: subgroup analysis	Moderate/severe ARDS vs. Control	0.80 (0.70-0.90)	62%	91%
	Mild ARDS vs. Control	0.67 (0.56-0.77)	44%	89%
	Moderate/severe ARDS vs. Mild ARDS	0.69 (0.55-0.83)	46%	91%
	Pulmonary vs. non-pulmonary ARDS	0.52 (0.37-0.67)	74%	40%
In-set: competing diagnoses	CPE vs. ARDS	0.65 (0.51-0.79)	58%	74%
	Pneumonia vs. ARDS	0.69 (0.49-0.88)	83%	64%
	CPE vs. Moderate/severe ARDS	0.76 (0.61-0.92)	74%	74%
	Pneumonia vs. Moderate/severe ARDS	0.76 (0.57-0.95)	91%	64%

### Temporal external validation

The eNose provided an ROC-AUC of 0.71 (CI: 0.54-0.87) in the temporal external validation cohort (Table 2). 27 of the 1000 random permutation tests resulted in a higher AUC-ROC, which means that chances of false discovery are 2.7%. A similar diagnostic accuracy was obtained with external validation using another eNose of the same manufacturer (AUC–ROC of 0.73 (CI: 0.58 – 0.90)).

### Subgroup analyses

The predicted probability of group membership by the eNose (result of logistic regression) was significantly different between moderate/severe ARDS, and mild ARDS (0.45 vs. 0.36, *P* = 0.01). The discrimination between moderate or severe ARDS and controls resulted in an AUC–ROC of 0.80 (CI: 0.70-0.90) with an optimal sensitivity of 91% and a specificity of 62%.

The predicted probability of group membership was not different between patients with a pulmonary (pneumonia, aspiration, etc.) and a non–pulmonary cause (sepsis, pancreatitis, etc.) for ARDS (0.41 vs. 0.38, *P* = 0.82).

### Competing diagnoses

The eNose signal was different between patients with pneumonia and patients with CPE from patients with ARDS, but with borderline significance levels (*P* = 0.05 and *P* = 0.05 vs. ARDS, respectively). Statistical significance and discrimination increased when patients with CPE and pneumonia were compared to patients with moderate/severe ARDS (*P* = 0.003 and *P* = 0.01; Table 2).

### Sensitivity analysis

The influence of co–variates on the association between exhaled breath and ARDS was assessed by comparing the log odds–ratio of the signal derived from the eNose (4.9 (CI: 2.5 - 7.6)) for ARDS in an unadjusted logistic regression model to the log odds-ratio found in a logistic regression model adjusted for the co–variate (Table 3).

**Table 3:** Sensitivity analysis for potential confounders

**Model**	**Log odds-ratio**	**P-value for eNose signal**
eNose unadjusted	4.9 (2.5-7.6)	0.0001
eNose + comorbidities	4.9	0.0001
eNose + PaO2/FiO2	3.6	0.0089
eNose + minute volume	4.9	0.0002
eNose + APACHE II	5.8	0.0002
eNose + SAPSII	5.7	0.0002

## Discussion

This study with a commercially available eNose suggests that breath analysis might be used to identify patients with ARDS if the eNose technology would mature towards this application with increased diagnostic accuracy and sensor stability. The diagnostic accuracy was good for moderate/severe ARDS. These findings were confirmed by temporal external validation. Notably, the exhaled breath profile from patients with CPE and pneumonia was well distinguished from that of patients with moderate/severe ARDS. These data support the suggestion that eNose assessment may qualify as a candidate test for future non-invasive diagnostic approaches of ARDS.

This is the first study to look at the diagnostic accuracy of an eNose for the diagnosis of ARDS. Earlier studies focussed on biological makers in broncho–alveolar lavage fluid or exhaled breath condensate [12,20,39-42]. These sampling methods are time–consuming and sample analysis is not available in the intensive care unit. A pioneer paper by Schubert *et al.* reported on gas–chromatography and mass–spectrometry of the exhaled breath in ARDS patients, thereby detecting specific compounds in the breath [13]. The concentration isoprene was reported to be significantly lower in the breath of ARDS patients; however, neither sensitivity nor specificity was given. The present data extend those results by providing the diagnostic accuracy of exhaled breath profiling.

The reported AUC-ROC of 0.71 provides moderate accuracy and is lower than previously found accuracies using the same type of eNose, when discriminating between other pulmonary diseases. For example, the externally validated diagnostic accuracy was 0.95 when discriminating between asthma and COPD [43]. Several explanations can be given. First, alterations of exhaled VOC patterns may not always occur during ARDS or do also occur in ICU patients without ARDS. Second, the index–test may not be sufficiently accurate, as may be suggested by sensors drift, even though we carefully dealt with that. Finally, the reference–test may not be perfect, which is not uncommon in diagnostic research [44].

The gold–standard is an inherent problem in current diagnostic research of ARDS. Indeed, the new ARDS definition was found to be 89% sensitive but only 63% specific for diffuse alveolar damage, the histological hallmark of ARDS [2,45,46]. This discordance was most profound in patients with mild ARDS. In the present study, we were not able to obtain the histo–pathological gold standard for ARDS. In general, lack of a gold–standard attributes to a lower observed diagnostic accuracy [44]. In our study, we found an increasing likelihood for correct classification with increasing severity of ARDS. Furthermore, there was no difference in discrimination between patients with a pulmonary and a non–pulmonary causal factor for ARDS. These findings are in line with the hypothesis of an imperfect reference standard and indirectly support the validity of exhaled breath analysis for the diagnosis of ARDS.

Patients with ARDS were discriminated from patients pneumonia and CPE with modest accuracy. However, differentiation between these disease states is regarded as one of the major clinical challenges in this patient population and in this scenario the eNose does not seem to provide answers. Diagnostic accuracy did increase when only patients with moderate/severe ARDS were regarded as cases, but was still moderate. Interestingly, the discrimination between moderate/severe ARDS and controls was profoundly sensitive whilst comparison to pneumonia was mostly specific. Thus ARDS can be excluded with confidence when compared to control subjects while it can’t be when compared to pneumonia patients. Possibly, some patients in the pneumonia group actually had ARDS but chest x-ray was too insensitive to detect the bilateral infiltrates. Alternatively, some patients with ARDS also had pneumonia and the differences in exhaled VOCs was just too small to separate these phenotypes adequately.

One of the strengths of this paper is the assessment of the external validity of the diagnostic algorithm. External validation is strongly recommended to limit false–discovery and over–fitting of diagnostic models [30]. Other strong points of this study may be represented by the recruitment of a relatively large number of patients, completely independent assessment of both index (exhaled breath analysis) and reference–test (ARDS diagnosis) and pre–defined subgroup analyses. Although the use of two ARDS definitions may seem a possible limitation we feel that we handled this carefully as all analyses were performed with the Berlin definition, which is more clearly defined with regards to disease severity and radiological criteria.

The implicit limitation of this study is that the VOCs altered in ARDS were not identified. This would require the use of gas–chromatography and mass–spectrometry (GC–MS), currently the best method for VOC–detection [47]. This is certainly required for understanding of the underlying pathophysiological pathways leading to altered VOC concentrations in the exhaled breath. VOC–identification was beyond the objective of the present study, because we aimed to establish diagnostic accuracy in the clinical setting. To that end, we performed sensitivity and specificity analysis based on composite VOC–signals, thereby taking maximal benefit of the multiple (as yet unknown) biomarkers involved. Therefore, eNose technology is adequate for testing hypotheses on diagnostic accuracy [25]. Diagnosis by eNose is rapid, cheap and easy to perform and therefore closer to clinical applicability than most other methods for exhaled breath analysis available at this moment.

Second, we cannot exclude that patient–related factors such as ventilation strategies, therapy, comorbidities and exposure to metabolic active compounds are (partly) responsible for the altered exhaled breath signal. However, sensitivity analyses showed that ventilator settings such as minute volume ventilation and comorbidity are probably not responsible for the found signal. It is difficult, if not impossible, to control for all confounders in an observational study but this can be accomplished in pre–clinical experiments. Importantly, lipopolysacharide–induced lung injury was found to induce changes in exhaled breath profiles in three separate experimental rat models [11,48]. It may very well be that a similar signal was detected in the present study, using a different analytical technique.

The prevalence of ARDS was high in the studied patient cohort, and higher than in most previous cohort studies. Several factors could serve as an explanation for this discrepancy. First, included patients were severely ill, as suggested by the high disease severity scores and the high mortality. Second, different from other cohorts of critically ill patients, we excluded patients after cardiopulmonary surgery. Finally, ARDS was assessed prospectively by a team of trained research fellows. Prospective assessment may identify patients that could have been missed retrospectively.

In this study, we used SPLS logistic regression analysis for model development. This algorithm can be used for variable selection in high dimensional datasets with low numbers of patients, while maintaining external validity and limiting false discovery [38]. Another advantage of SPLS is that the produced model is relatively simple to interpret. Some sensors were found to be predictive of ARDS when the sensor result was lower compared to control, as indicated by a negative coefficient in the model (Table 2). This is probably due to lower breath concentrations of VOCs with affinity to these sensors. Following GC–MS driven research, we can hypothesize that isoprene can be one of these VOCs [13]. Interestingly, not all coefficients were negative: apparently three sensors are affinitive for VOCs that increase in concentration. Combined, these findings provide evidence that ARDS is associated with both up–regulation and down–regulation of volatile metabolites.

This paper describes exhaled breath analysis as a diagnostic tool for ARDS. However, several steps need to be taken before exhaled breath analysis can be implemented into clinical practice. Primarily, we need a list of potential ARDS–biomarkers in exhaled air obtained from controlled pre–clinical models, thereby excluding confounders as medication, comorbidities and ventilatory strategies. Second, the accuracy of sensors needs to be increased as the tested commercially available technology proved insufficient: sensor sensitivity and specificity for VOCs can be modified targeting potential biomarkers. Drift should be minimized and sensor-arrays should provide interchangeable results to allow for application in large clinical trials. Continuous exhaled breath analysis would allow for monitoring. Finally, the lack of a gold standard cannot be solved easily. In the long run, a move from the diagnostic accuracy paradigm towards a test validation paradigm might be justified [44]. This would allow for the comparison of added value of several index–tests, including exhaled breath analysis, in clinical decision–making.

## Conclusions

We found that an electronic nose can rapidly and non–invasively discriminate between patients with and without ARDS with modest accuracy. The diagnostic model was both externally validated and reproducible. Diagnostic accuracy increased when only moderate and severe ARDS patients were considered. The exhaled breath profile from patients with CPE and pneumonia was different from that of patients with moderate/severe ARDS.

## Competing interests

The authors declare that they have no competing interests.

## Authors’ contributions

LDJB participated in the design of the study, collected and analyzed the data and drafted the manuscript. MJS participated in the design of the study, supervised the data analysis and revised the manuscript. PJS conceived the study, participated in its design and revised the manuscript. All authors approved the final version of the manuscript.

## Pre-publication history

The pre-publication history for this paper can be accessed here:

http://www.biomedcentral.com/1471-2466/14/72/prepub

## Supplementary Material

Additional file 1 Exhaled Breath Profiling for Diagnosing ARDS in Intubated and Ventilated ICU–Patients. Click here for file
